# Videoconferencing psychotherapy: determining acceptance, drivers and barriers of use

**DOI:** 10.3389/fdgth.2025.1634013

**Published:** 2025-08-29

**Authors:** Angelina Nurtsch, Lisa Maria Jahre, Julia Barbara Krakowczyk, Anita Robitzsch, Martin Teufel, Alexander Bäuerle

**Affiliations:** ^1^ Clinic for Psychosomatic Medicine and Psychotherapy, LVR-University Hospital, University of Duisburg-Essen, Essen, Germany; ^2^ Center for Translational Neuro- and Behavioral Sciences (C-TNBS), University of Duisburg-Essen, Essen, Germany

**Keywords:** eHealth, telemedicine, remote consultation, UTAUT, mental health

## Abstract

**Background:**

With increasing digitalization in psychotherapy, some healthcare interactions are transitioning to online services. This study examined the acceptance of videoconferencing psychotherapy (VCP) among patients affected by mental health disorders and healthy controls, identifying drivers and barriers.

**Methods:**

A cross-sectional survey study was conducted from February to October 2024 in North Rhine-Westphalia, Germany. Participants were recruited via outpatient clinics, online study platforms, and psychotherapy-related social media. Inclusion criteria were age ≥ 18 years, German language proficiency, and internet access. Sociodemographic, medical, psychotherapeutic anamnesis, and information and communication technologies (ICT) related data were collected. Acceptance of VCP was assessed using an extended Unified Theory of Acceptance and Use of Technology (UTAUT) model.

**Results:**

Of *N* = 483 participants, 47.6% (*n* = 230) reported high, 34.2% (*n* = 165) moderate and 18.2% (*n* = 88) low acceptance. Significant predictors included digital overload (*β* = .14, *p* = .006), depressive symptoms (*β* = .11, *p* = .033), current psychotherapy: outpatient (*β* = -.34, *p* = .003), concern: effectiveness (*β* = -.47, *p* < .001), concern: emotional expression (*β* = -.25, *p* < .001), and the UTAUT predictors: social influence (*β* = .28, *p* < .001), performance expectancy (*β* = .32, *p* < .001) and effort expectancy (*β* = .15, *p* = .001). Explained variance of the final model was 72.9%.

**Conclusions:**

The moderate to high acceptance indicates that VCP could supplement psychotherapeutic care addressing the global treatment gap. Identified drivers and barriers highlight factors that should be considered to enable broader implementation.

## 1 Introduction

The most recent “World mental health report” published in 2022 by the World Health Organization (WHO) indicates a rising prevalence of mental health issues worldwide over the past 20 years, driven by global crises such as pandemics, humanitarian emergencies, armed conflicts, and climate change (1–7). Concurrently, the global treatment gap is expanding, as mental health care systems in numerous countries are inadequately resourced to meet patients' needs (7). Complementing these findings, the latest “Ipsos health service report” from 2024, based on responses from 31 countries, identifies mental health as the most critical health concern globally. The report highlights limited access to care and prolonged waiting times as key challenges within the overall healthcare system (8). Further insights from the more specific “Ipsos world mental health day report” from 2024 reveal that 62% of global respondents have experienced stress to an extent that negatively affected their daily lives at least once. The prevalence of such stress-related impairment varies significantly between countries, ranging from 76% in Turkey to 44% in Japan. Moreover, the report emphasizes that these impairments can be associated with broader socio-economic consequences, including reduced productivity and increased time off work (9).

In Germany, securing an outpatient psychotherapy appointment remains challenging, with demand doubling over the past 20 years, according to the German Federal Chamber of psychotherapists (10). Despite the implementation of the Appointment Service and Care Act (TSVG) in Germany in 2019, which aimed to improve the appointment process, patients still face considerable waiting times, with an average waiting period of three to nine months, as this also presents itself as a global problem (7, 11–13). To enhance global patient care, optimize service delivery, and overcome access barriers, expanding location-independent videoconferencing psychotherapy (VCP) as a complement to traditional face-to-face psychotherapy should be considered (14).

Digitalization is rapidly transforming the global healthcare sector, with telemedicine becoming increasingly significant (14). Driven by the COVID-19 pandemic and limited access to in-person care, video consultations have become an important part of medical and psychological care (15–23). Telemedicine facilitates healthcare delivery across physical distances through information and communication technologies (ICT), such as computers and mobile phones, thereby improving accessibility and efficiency of patient care (24, 25). In Germany, several laws, including the “eHealth law” in 2015, have been enacted to facilitate and expand the use of telemedicine (26).To further advance its integration, especially in psychotherapeutic context, the German Bundestag introduced the “Digital-Law – DigiG” in 2023, eliminating previous limitations in the quantity of VCP sessions and providing the necessary legal framework (27, 28). The international legal background shows similar diversity. Selected examples include the United States, where guidance from the American Psychological Association (APA) and the American Telemedicine Association (ATA) shapes ethical and clinical practice, and Australia, where the Australian Psychological Society (APS) issues professional standards (29, 30). By contrast, countries such as Saudi Arabia remain in the early stages of developing comprehensive, telepsychology-specific regulatory frameworks (31).

Telemedicine, particularly tele-psychotherapy including VCP, can facilitate access to psychotherapy by overcoming geographical and mobility barriers (14, 32, 33). Beyond the increased convenience of home-based therapy, it may reduce costs and time related e.g., to travel, time off work, and childcare as demonstrated by various studies on the advantages of telemedicine (14, 21, 23, 33–37). Telemedicine can thus improve access to psychotherapy, particularly for older or mobility-restricted patients and those in rural areas (14, 21, 23, 33). Additionally, tele-psychotherapy has the potential to redistribute patients from underserved regions to available mental health professionals, thereby optimizing the utilization of treatment capacity (14, 38). This potential is further highlighted by a review of 23 international studies, including research from Australia, the United States, Canada, Scotland, and the United Kingdom and a study on tele-mental health services for indigenous peoples in Northern Quebec (38, 39). Thus VCP may contribute to improve treatment outcomes and quality, as well as patients' quality of life through the effective use of tele-mental health services (14).

Previous research on acceptance and satisfaction of VCP highlights several benefits especially of this technology, including a more comfortable and less threatening environment than face-to-face therapy (20). Patients may be more likely to engage in VCP due to an increased sense of security (19). Moreover, its neutral therapeutic setting enhances patients' sense of control and may reduce stigma-related concerns—a significant barrier to seek psychotherapy (22, 38). This is also supported by a recent study from Germany, which shows that the use of tele-mental health services was not only associated with the desire to avoid stigmatization, but also with full-time employment, long waiting times and an inconvenient appointment scheduling, highlighting the importance of service-related advantages including flexibility, accessibility, and reduced barriers to care (40).

Despite its advantages, several challenges concerning the VCP implementation remain, including concerns about data protection and limited technical readiness among mental health care providers (40). Studies have also identified technical difficulties, like the absence of reliable broadband internet, as a significant obstacle, interrupting the communication between therapist and patient (41). Therapists may experience a certain sense of isolation within the therapeutic relationship due to the physical distance, which may also affect their patients (32). These challenges may increase due to the limited eye contact and the restricted visibility of participants' posture on the screen (21, 41, 42). However, this non-verbal behavior is essential for establishing a therapeutic relationship and the accurate recognition and interpretation of the patient's emotions (23). Moreover, expressing empathy or providing comfort through non-verbal cues can be particularly challenging in a VCP setting (32, 42). Several studies also highlight the increased risk of distractions at home, including incoming e-mails or family interruptions, which may further reduce VCP quality (21, 41).

Although there are barriers which must be overcome for a successful implementation, VCP presents significant advantages, as shown above, that may address existing needs for optimized mental health care. Consequently, it is essential to evaluate the acceptance of these services in advance, as this may indicate their future utilization. Furthermore, identifying both the drivers and potential barriers to adoption is therefore important (43). The Unified Theory of Acceptance and Use of Technology (UTAUT) is particularly suitable for evaluating acceptance and use of eHealth interventions (44–51). The UTAUT model emphasizes three key predictors which affect acceptance, operationalized as behavioral intention (BI), and the actual usage of a technology. These predictors include performance expectancy (PE), referring to the perceived benefits of the technology; effort expectancy (EE), denoting its perceived ease of use; and social influence (SI), reflecting the impact of the user's personal environment (including family or healthcare providers) on the use of tele-psychotherapy (45, 49, 50).

The acceptance of VCP has been subject of increasing international research, highlighting its significance in addressing the global mental health treatment gap (52–55). As this study focuses on the context of psychotherapy in Germany, a more detailed comparison with international findings is provided in the discussion.

### 1.1 Objectives

The aim of this study is to assess the acceptance of VCP among patients affected by mental health disorders and healthy controls, acknowledging that some controls might have undiagnosed symptoms or may seek psychotherapy in the future. In light of the global treatment gap in mental health care, the study also aimed to evaluate VCP as a valuable supplement to in psychotherapeutic care. Beyond general acceptance, it is important to determine whether participants would be willing to actually use this technology and to what extent, in order to assess whether it should be implemented on a broader scale. Furthermore, it is essential to identify both the drivers and barriers to VCP acceptance, as well as concerns regarding its use to optimize its adoption and practical implementation. To identify further predictors of acceptance, an extended version of the UTAUT model has been developed by incorporating sociodemographic, medical, psychotherapeutic anamnesis and ICT-related data. It is important to determine to what extent acceptance of VCP and subsequent behavioral intention depend on these individual factors, in order to specifically address and promote them – so that everyone can benefit from these digital solutions.

## 2 Methods

### 2.1 Study design

A cross-sectional online-based open survey study was conducted to examine the acceptance of VCP, and its drivers and barriers among patients affected by mental health disorders and healthy controls. The survey was designed by experts from the fields of medicine, psychology and eHealth, containing seven pages with a maximum of 19 items per page. To evaluate its functionality and usability, various uninvolved colleagues tested it prior to its use. No adaptive questioning was used due to the low complexity of the questions, and participants were not allowed to review or change their answers after continuing to the next page to ensure response integrity. Items were presented in their validated order. Data collection took place from February to October 2024 via the platform Unipark (TIVIAN GmbH), with an average completion time of *M* = 12.5 (*SD* = 6.8) minutes (56). Unusual completion times were monitored, but no participants were excluded based on this criterion. The survey was initially started by *N* = 574 participants, of which 80.49% (*N* = 462) completed it. Due to not fulfilling the inclusion criteria, *n* = 33 participants had to be excluded. Further, *n* = 58 participants were excluded because of missing values on the primary outcome (acceptance). Therefore, *N* = 483 participants were included in the final data analysis. To maintain the required high methodological standards, the Checklist for Reporting Results of Internet E-Surveys (CHERRIES (57);) was applied (see Supplementary Table SM1).

### 2.2 Study population

The sample included both individuals currently affected by mental health disorders and healthy controls, acknowledging that some controls might have undiagnosed symptoms or may seek psychotherapy in the future. Participants were recruited from February to October 2024 from various regions across Germany. Recruitment took place in psychotherapeutic outpatient clinics and practices in North Rhine-Westphalia (Germany) via study distribution networks, as well as psychotherapy-related social media channels and blogs. Study information was distributed via flyers, posters, social media posts and the study staff and contained information on its purpose, conductors, estimated completion time and anonymity. Inclusion criteria were legal age (≥18 years), good knowledge of the German language, and Internet access. Before the beginning of the survey, digital informed consent was given. Participation was voluntary, anonymous, and without compensation or financial incentive.

### 2.3 Assessment instruments

The survey was composed of sociodemographic, medical, psychotherapeutic anamnesis, and ICT-related data (including acceptance). Validated assessment instruments and self-generated items were used to collect responses. The questionnaire is included in the Supplementary Materials.

The first section of the survey assessed sociodemographic variables, including age, gender, marital status, population size of their place of residence, level of education, and occupational status. Additionally, participants stated whether an illness affected their mobility (e.g., going to the doctor).

Subsequently, psychotherapeutic anamnesis data was obtained, which inquired about the presence of any diagnosed mental health disorder. Participants were also asked about their mental health treatment (e.g., psychiatric outpatient clinic, psychiatric/psychosomatic inpatient psychotherapy, outpatient psychotherapy, psychiatric medication, socio-psychiatric service). Further, participants indicated the frequency of their outpatient psychotherapy sessions per month. The Patient Health Questionnaire Scale-8 (PHQ-8) was utilized to assess depressive symptoms over a two-week period using a four-point Likert scale (0 = “never” to 4 = “almost every day”) (58). Internal consistency was high (Cronbach's *α* = .89).

In the following section of the survey, respondents were asked to report their general ICT usage on a five-point Likert scale, covering aspects such as digital confidence (47, 48, 59–62), internet anxiety (47, 48, 59–62), experiences of digital overload (47), as well as the use of Internet and VC for personal and professional purposes (46). Digital confidence was assessed in relation to the use of digital media, end devices, as well as internet and VC platforms (1 = “very uncertain” to 5 = “very safe”). Internal consistency was excellent (Cronbach's *α* = .93). Respondents could indicate their daily internet and VC usage on a scale from 1 (= “not at all”) to 5 (= “more than five hours per day”). Internet anxiety (e.g., “I have concerns about using the internet”) and digital overload (e.g., “I feel burdened by the constant availability via phone or email.”) were measured with three items each and rated on a five-point Likert scale (1 = “strongly disagree” to 5 = “strongly agree”). Internal consistency was good for both constructs (Internet anxiety: Cronbach's *α* = .76; digital overload: *α* = .72). Participants' existing knowledge of digital health services in psychotherapy was assessed using three items, rated on a five-point Likert scale (1 = “strongly disagree” to 5 = “strongly agree”) (46). Internal consistency was high (Cronbach's *α* = .81).

The revised German version of the eHealth Literacy Scale (GR-eHEALS) was used to evaluate respondents' digital health literacy (63). Eight items were rated on a five-point Likert scale (1 = “strongly disagree” to 5 = “strongly agree”), enabling respondents to indicate e.g., their knowledge regarding how to find useful health information online, as well as their ability to critically evaluate and effectively use it. Sum scores range from 8 to 40, with higher scores indicating greater levels of eHealth literacy. Internal consistency was good (Cronbach's *α* = .72).

Participants were asked whether their current psychotherapist, if they were in treatment, already offered VCP, if they had utilized it, and how frequently. Additionally, the context in which they would use VCP (e.g., first consultation, medical prescriptions, or outpatient psychotherapy) was assessed, along with the percentage of psychotherapy sessions they would conduct via video. Concerns that might hinder VCP usage (e.g., technical issues, data protection, difficulties in emotional expression or non-verbal communication) were assessed using a five-point Likert scale (1 = “strongly disagree” to 5 = “strongly agree”). Furthermore, participants rated the importance of prior in-person meetings and establishing a relationship of trust beforehand on a five-point Likert scale (1 = “very important” to 5 = “not important at all”).

Acceptance of VCP was assessed using the UTAUT model, with acceptance operationalized as BI measured by three items: “I would like to try videoconferencing psychotherapy”, “I would use videoconferencing psychotherapy if it was offered to me” and “I would recommend videoconferencing psychotherapy to acquaintances with a mental disorder” (44). Internal consistency of this scale was high (Cronbach's *α* = .88). The three key predictors of the UTAUT model (SI, EE, PE) were also examined. SI was assessed using two items (e.g., “People close to me would approve my usage of videoconferencing psychotherapy”). Internal consistency was good (Cronbach's *α* = .68). EE was measured with five items (e.g., “Videoconferencing psychotherapy would be easy for me to use and understand”). Internal consistency was good (Cronbach's *α* = .76). PE was evaluated with four items (e.g., “Using videoconferencing psychotherapy could improve my mental health status”). Internal consistency was good (Cronbach's *α* = .78). Responses were given on a five-point Likert scale (1 = “strongly disagree” to 5 = “strongly agree”).

### 2.4 Statistical analyses

Statistical analysis was conducted using R (4.3.1) and RStudio. Sum score was calculated for the PHQ-8. Mean scores were computed for GR-eHEALS, digital confidence, digital overload, internet anxiety, and prior knowledge. Further, mean scores for acceptance (= BI) and its three predictors PE, EE, and SI were calculated. BI was then divided into three categories in accordance with previous research (48, 64): Low (scores from 1 to 2.34), moderate (scores from 2.35 to 3.67) and high acceptance (scores from 3.68 to 5). Descriptive statistics were applied for sociodemographic, medical, psychotherapeutic anamnesis and ICT-related data. Multiple hierarchical regression analysis was conducted to examine the drivers and barriers of acceptance of VCP. Predictors were included blockwise: (1) sociodemographic data, (2) psychotherapeutic anamnesis, (3) ICT-related data, (4) concerns of VCP, and (5) UTAUT predictors. The generalized variance inflation factors (GVIF) indicated no multicollinearity (all GVIF values < 2.4). Visual inspection of Q-Q-plots of the residuals showed no signs of violations against normality. A scatter plot of the standardized residuals and the adjusted predicted values verified homoscedasticity. The level of significance was set to *α* < .05 for all tests.

### 2.5 Ethics of the study

The conductance of the study was approved by the Ethics Committee of the Medical Faculty of the University of Duisburg-Essen (19–89-47-BO).

## 3 Results

### 3.1 Study population

Among the *N* = 483 participants, the mean age was *M* = 35.22 (*SD* = 13.35) years. The youngest participant was 18 years old and the oldest was 92 years old. The majority of participants were identified as female (*n* = 373, 77.2%; male: *n* = 105, 21.7%; diverse: *n* = 5, 1.0%). Of the sample, 30.2% (*n* = 146) were diagnosed with one mental disorder, while 25.5% (*n* = 123) were affected by multiple mental disorders. Regarding general health, 20.5% (*n* = 99) of the participants reported an illness that affected their mobility. Outpatient psychotherapy was currently utilized by 26.0% (*n* = 126). Among these outpatients, the average number of therapy sessions per month was *M* = 3.07 (*SD* = 2.09). The average PHQ-8 score, indicating depressive symptoms, was *M* = 8.57 (*SD* = 5.75). Table 1 shows additional characteristics of the study sample.

**Table 1:** Sample characteristics.

Variables	*N* (%)	*M (SD)*
Age		35.22 (13.35)
Gender		
Female	373 (77.2)	
Male	105 (21.7)	
Diverse	5 (1.0)	
Marital status		
Single	177 (36.6)	
In a relationship	151 (31.3)	
Married	115 (23.8)	
Divorced/separated	28 (5.8)	
Widowed	3 (0.6)	
Other	9 (1.9)	
Educational level		
No educational degree/Other	8 (1.6)	
Lower secondary education	22 (4.6)	
Higher secondary education	72 (14.9)	
Higher education entrance qualification	205 (42.4)	
University education	176 (36.4)	
Occupational status		
Still in education (e.g., school, university)	135 (28.0)	
Not employed (e.g., job-seeking, unfit to work)	40 (8.3)	
Part-time employed	99 (20.5)	
Employed	161 (33.3)	
Retired	21 (4.3)	
Other	27 (5.6)	
Currently unfit to work	77 (15.9)	
Place of residents (population size)		
Large city (>100,000 residents)	317 (65.6)	
Medium sized city (>20,000 residents)	84 (17.4)	
Small town (>5,000 residents)	41 (8.5)	
Rural area (<5,000 residents)	41 (8.5)	
Current mental health treatment		
Psychiatric outpatient clinic (institutional setting)	61 (12.6)	
Psychiatric inpatient psychotherapy	8 (1.7)	
Psychosomatic inpatient psychotherapy	9 (1.9)	
Outpatient psychotherapy	126 (26.1)	
Psychiatric medication	108 (22.4)	
Socio-psychiatric service	12 (2.5)	
**Total**	483 (100.0)	

### 3.2 ICT-related data and responses towards videoconferencing psychotherapy

The majority of participants (76.6%, *n* = 370) had never used VCP before. Those who had prior experience with it had used VCP *M* = 5.14 (*SD* = 3.46) times before. Participants indicated that they would be willing to conduct *M* = 49.89% (*SD* = 31.94%) of psychotherapy sessions via video. In general, participants rated their need of personal trust before use of VCP as rather important (*M* = 1.81, *SD* = 1.09, range 1–5). Among the participants, 78.74% (*n* = 363) found personal trust “very/rather important”, while only 10.62% (*n* = 49) stated personal trust as ‘very/rather unimportant‘.

**Figure 1:**
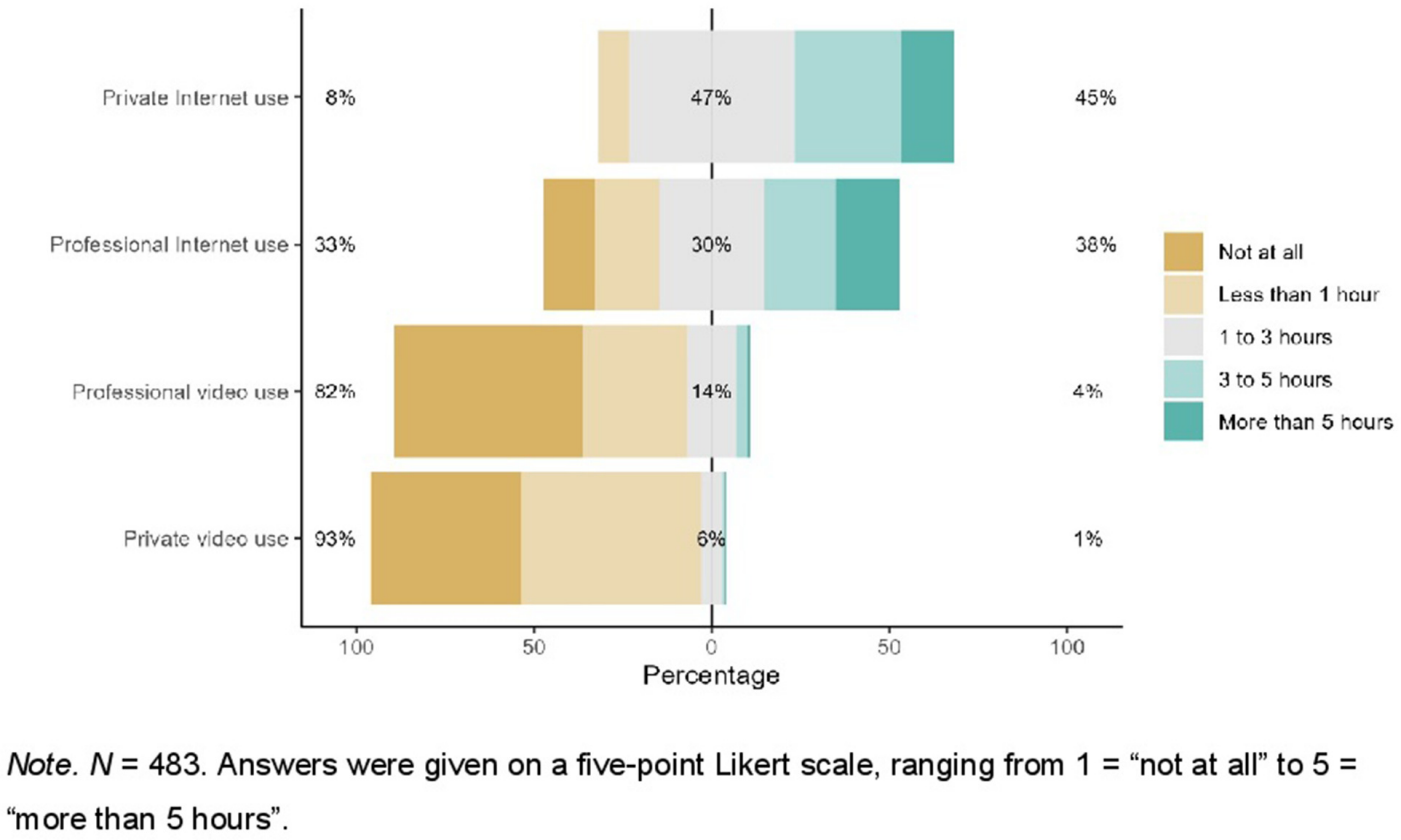
Use behavior of the internet and videoconferencing for private and professional purposes.

**Figure 2:**
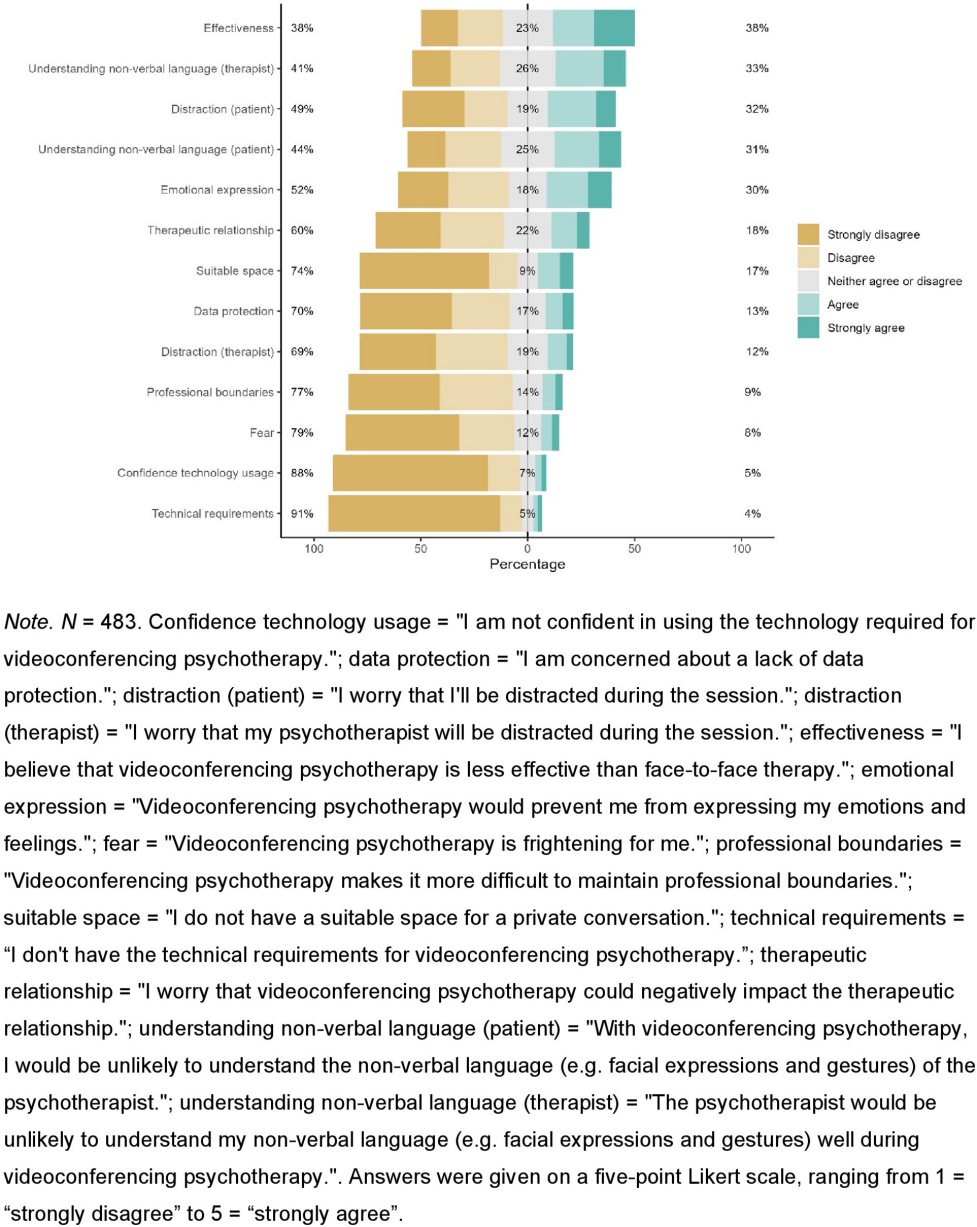
Concerns regarding videoconferencing psychotherapy.

Further, the participants of this study reported a high level of digital confidence (*M* = 4.19, *SD* = 0.89), moderate digital overload (*M* = 2.55, *SD* = 0.9) and low Internet anxiety (*M* = 1.59, *SD* = 0.69). Prior knowledge about any digital approaches of psychotherapy was above average (*M* = 3.24, *SD* = 1.05). Participants indicated high levels of eHealth literacy (*M* = 32.40, *SD* = 6.00, range 8–40). Use behavior of the internet and videoconferencing for private and professional purposes is visualized in Figure 1. Participants responses towards VCP are summarized in Table 2. Potential concerns regarding VCP are visualized in Figure 2.

**Table 2:** Responses towards videoconferencing psychotherap*y.*

Variables	*N* (%)
Informed about VCP	
Yes	207 (42.9)
No	254 (52.6)
NA	22 (4.6)
Availability of VCP at psychotherapist	
Yes	84 (17.4)
No	58 (12.0)
I don't know	77 (15.9)
Not in treatment (currently or in the past)	242 (50.1)
NA	22 (4.6)
Potential use of VCP for	
First consultation	217 (44.9)
Pharmacological adjustment	255 (52.8)
Medical prescriptions/certificate of incapacity for work	289 (59.8)
Outpatient psychotherapy	271 (56.1)
Inpatient psychotherapy	17 (3.5)
Partial inpatient psychotherapy	47 (9.7)
Psychiatric outpatient clinic (institutional setting)	86 (17.8)
Final consultation	135 (28.0)
I wouldn't use digital psychotherapy via video for any of these purposes.	30 (6.2)
NA	22 (4.6)
**Total**	483 (100.0)

NA, data not available; VCP, videoconferencing psychotherapy.

### 3.3 Acceptance of videoconferencing psychotherapy and its predictors

Overall, acceptance of VCP was moderate to high (*M* = 3.63, *SD* = 1.14). High acceptance was reported by 47.6% (*n* = 230). Moderate acceptance was indicated by 34.2% (*n* = 165), while 18.2% (*n* = 88) reported low acceptance.

Multiple hierarchical regression analysis was conducted to determine predictors of acceptance of VCP. Due to missing data on the predictor variables, *n* = 44 participants had to be excluded to ensure the necessary participant number per category.

Sociodemographic data were included in the first step [*R*^2^ = .015, *R*^2^*_adj_* = .004, *F*(5,433) = 1.35, *p* = .241]. The explained variance of the first step was 1.5%. There were no significant predictors of acceptance in this step.

In the second step, psychotherapeutic anamnesis data were included [*R*^2^ = .039, *R*^2^*_adj_* = .023, *F*(7,431) = 2.50, *p* = .016], which significantly increased the explained variance to 3.9% [*ΔR*^2^ = .024, *F*(2,431) = 18.35, *p* < .001]. *Depressive symptoms* (*β* = .11, *p* = .033) and *Outpatient psychotherapy (currently)* (*β* = -.34, *p* = .003) were significant predictors of acceptance.

ICT-related data, included in the third step [*R*^2^ = .064, *R*^2^*_adj_* = .042, *F*(10,428) = 2.93, *p* = .001], significantly increased the explained variance to 6.4% [*ΔR*^2^ = .025, *F*(3,428) = 12.92, *p* < .001]. *Digital confidence* was a significant predictor of acceptance (*β* = .14, *p* = .006).

In the fourth step, concerns of VCP were included [*R*^2^ = .506, *R*^2^*_adj_* = .488, *F*(15,423) = 28.86, *p* < .001]. The explained variance significantly increased to 50.6% [*ΔR*^2^ = .442, *F*(5,423) = 137.01, *p* < .001]. *Concern: effectiveness* (*β* = -.47, *p* < .001) and *Concern: emotional expression* (*β* = -.25, *p* < .001) were significant predictors of acceptance.

The three UTAUT predictors were included in the final step [*R*^2^ = .729, *R*^2^*_adj_* = .718, *F*(18,420) = 62.82, *p* < .001]. Explained variance of the final model was significantly increased to 72.9% [*ΔR*^2^ = .223, *F*(3,420) = 115.46, *p* < .001]. *SI* (*β* = .28, *p* < .001), *PE* (*β* = .32, *p* < .001) and *EE* (*β* = .15, *p* = .001) were significant predictors of acceptance of videoconferencing psychotherapy. Table 3 contains the final UTAUT model of acceptance and its predictors.

**Table 3:** Results of the multiple hierarchical regression analysis of acceptance towards videoconferencing psychotherapy.

Predictors	*B*	*β*	*t*	*R^2^*	*ΔR^2^*	*p*
(Intercept)	.52	-.07	1.30			.195
Step 1: Sociodemographic data				.015	.015	
Age	-.00	-.02	−0.81			.418
Female gender	.11	.10	1.51			.132
Place of residence: Medium sized city (>20,000 residents)	-.03	-.03	-.38			.701
Place of residence: Small town (>5,000 residents)	.01	.01	.07			.944
Place of residence: Rural area (<5,000 residents)	-.14	-.12	−1.34			.182
Step 2: Psychotherapeutic anamnesis				.039	.024	
Depressive symptoms (PHQ-8)	.01	.06	2.09			.038
Outpatient psychotherapy (currently)	.02	.02	0.31			.759
Step 3: ICT-related data				.064	.025	
Digital confidence	.07	.06	1.93			.054
Internet anxiety	.09	.05	1.86			.064
eHealth literacy	-.07	-.04	-1.59			.113
Step 4: Concerns				.506	.442	
Effectiveness	-.15	-.18	−4.37			<.001
Understanding non-verbal language (therapist)	.01	.01	0.11			.910
Distraction (patient)	.01	.02	0.46			.644
Understanding non-verbal language (patient)	.00	.00	0.06			.949
Emotional expression	-.11	-.13	−3.21			.001
Step 5: UTAUT predictors				.729	.223	
Social influence	.34	.28	8.33			<.001
Performance expectancy	.40	.32	8.08			<.001
Effort expectancy	.21	.15	3.41			.001

*N* = 439. To examine the drivers of acceptance towards videoconferencing psychotherapy, operationalized as behavioral intention, multiple hierarchical regression analysis was applied. Drivers were included blockwise in four steps. In Step 2, 3 and 4 only the newly included variables are presented. *B* = Unstandardized beta. β = Standardized beta. *t* = Test statistic. *R*^2^ = Determination coefficient. *ΔR*^2^ = Changes in *R*^2^. PHQ-8 = Patient Health Questionnaire Depression Scale; UTAUT = Unified Theory of Acceptance and Use of Technology.

## 4 Discussion

The aim of the study was to assess the acceptance of VCP, its drivers and barriers among patients affected by mental health disorders and healthy controls, and to examine whether VCP could serve as a valuable supplement to overcome the global treatment gap in mental health care. Although some studies have examined VCP acceptance from the perspectives of therapists or patients, the majority were conducted during or prior to the COVID-19 pandemic—a period when many therapeutic interactions were necessarily transitioned to digital platforms (51, 65–70). To our knowledge, there is a lack of valid data on the drivers and barriers to VCP acceptance, particularly regarding concerns about its use in the post-pandemic context, when normalcy has returned to therapeutic contexts and face-to-face interactions are once again feasible.

Overall, respondents demonstrated a moderate to high level of acceptance towards VCP. Consistent with findings from previous studies on VCP acceptance, nearly half of the participants reported a high level of acceptance, while approximately one-third expressed moderate acceptance (66, 71). A systematic review of 39 studies conducted across 19 countries on the acceptability and usability of tele-mental health services supports the moderate to high satisfaction and acceptance of VCP found in this study. The review highlights the widespread uptake of these services particularly among adolescents, underscoring the potential for future growth and expansion of these interventions (72). Comparable levels of acceptability for eHealth interventions have also be observed in other medical domains, including oncology, cardiology, chronic pain management, obesity treatment, and general practice settings (46–48, 59, 61, 62, 64). By contrast, studies prior to the COVID-19 pandemic reported low to moderate acceptance to internet-based mental health services (69). This shift in acceptance may be attributed to the widespread adoption of eHealth services and telemedicine during the pandemic, which likely influenced perceptions and familiarity with these interventions (1, 66). Various drivers and barriers influencing acceptance were identified and are discussed below.

In contrast to other studies examining the acceptance of eHealth services in several medical fields, which identified e.g., younger age and higher education as significant predictors, sociodemographic parameters did not significantly influence VCP acceptance in the present study (47, 62, 67, 69, 73). These findings may also be attributed to the recent pandemic, which likely increased the overall acceptance of digital mental health services due to increased experience, regardless of sociodemographic factors, but also to the respondents' high eHealth literacy in general (1, 66, 74). Situational variables, like acute need for therapy or long waiting times for in-person treatment, may therefore have a greater influence on acceptance than sociodemographic variables. These results are in line with a recent study from Germany, which similarly found no significant association between the socioeconomic status and satisfaction with tele-mental health services. However, the study reports lower satisfaction and thus acceptance with telephone-only services among individuals in full-time employment with higher educational levels, suggesting that this group may have greater technological affinity and higher expectations, which in turn could indirectly influence satisfaction and, ultimately, VCP acceptance (75).

Regarding the psychotherapeutic anamnesis, present depressive symptoms and current outpatient psychotherapy emerged as significant predictors of acceptance. These findings align with prior research, which identified a correlation between mental health status and acceptance of e-mental health interventions (46, 48, 59, 76, 77). A possible explanation is that seeking psychotherapy might be associated with stigmatization, which could be alleviated by VCP, as it offers discreet participation from home (23, 33, 76, 78). VCP may also provide a convenient and non-threatening option for delivering psychological support to patients who might otherwise face barriers to therapy due to their condition (76). Patients currently undergoing outpatient psychotherapy may be more likely to seek therapy in general and then use VCP, driven not only by experiences of stigma but also by logistical challenges associated with face-to-face therapy, which could be mitigated by the convenience and accessibility of VCP (19, 33, 38). However, other studies suggest, that the online setting may feel less safe and comfortable, making it difficult to reveal the internal world (79). This contrast highlights that the perception of VCP can vary depending on individual preferences, experiences and especially psychological needs.

ICT-related data were also revealed as relevant influencing factors in this study. Digital confidence, as a significant driver of acceptance, highlights the importance of trust in ICT, which can substantially influence the willingness to engage with technologies like VCP (62, 80). The high levels of digital confidence observed provide a promising foundation for further implementation of VCP into routine care. Internet anxiety, as a general concern about internet usage, was reported as low, whereas digital overload, defined as a state of being overwhelmed due to continuous digital availability, was revealed as moderate (81, 82). Although these variables did not achieve statistical significance in this study, their relevance to acceptance remains noteworthy, contrasting with other studies on eHealth acceptance in which higher Internet anxiety or digital overload are typically associated with reduced acceptance of VCP and decreased eHealth utilization (47, 48, 60). Respondents reported frequent internet use in both personal and professional contexts, consistent with their high digital confidence and low internet anxiety—factors that may already contribute to their acceptance of eHealth interventions. The moderate digital overload suggests that digital interactions do not pose a significant burden, making it an unlikely barrier to VCP acceptance. Overall, these findings align with participants' high eHealth literacy, reflecting their ability to find and utilize digital health information which may directly enhance acceptance of VCP (46).

Two primary concerns regarding VCP were identified: effectiveness of VCP and the ability to convey emotional expression. Concerns about reduced effectiveness of VCP have been documented in previous research, thereby suggesting a lack of familiarity with or preconceived biases against VCP, where face-to-face therapy may still be regarded as standard (23). Empirical studies assessing its actual efficacy could demonstrate a comparable effectiveness and patients' satisfaction to face-to-face therapy (6, 83, 84). Furthermore, the therapeutic alliance, as an important predictor of treatment success, has been shown to be achievable in video-based formats (38, 66, 84–86). The implementation of targeted information to enhance public understanding of VCP and offering the opportunity to try out VCP before committing to this modality, could address this concern (6). Moreover, providing studies that substantiate the effectiveness of VCP could further enhance its acceptance. Concerns regarding hindered emotional expression suggest that digital interactions may be perceived as less “human”, with impaired nonverbal communication, which could be addressed by advancements in technology and, importantly, by targeted trainings for therapists, as emphasized by research on the working experience of mental health professionals (6, 19, 23, 41, 87). These findings also align with previous studies, where emotional distance was perceived as either disruptive and impersonal or helpful in opening up to the therapist. Expectations regarding emotional connection and communication quality particularly influenced acceptance, suggesting that such concerns are perceived differently depending on individual needs (66). Moreover, the interpersonal dimension of feeling understood and seen may be perceived differently via video, highlighting the need for therapists to develop specialized competencies in nonverbal communication especially since the therapeutic relationship represents an important factor in psychotherapy (6).

Although acceptance among respondents was moderate to high, these findings suggest that VCP is not universally applicable to all situations or patients, as participants reported willingness to conduct approximately 50% of their psychotherapy sessions via video. The majority expressed readiness to use VCP for specific purposes like medical prescriptions, issuing certificates of incapacity for work, outpatient psychotherapy, or pharmacological adjustments. In contrast, only a few considered it suitable for inpatient psychotherapy, and around 6% would not use VCP for any of the scenarios presented. This aligns with findings from a systematic review from 2022, which indicated that VCP appears more suitable for less complex or stable cases, whereas emotional crises were considered more difficult to address due to challenges in nonverbal communication—matching the concerns identified in the present study (66, 74). Establishing a foundation of personal trust with the therapist was identified as an important prerequisite for using VCP, underlining the importance of the practitioner-patient relationship in psychotherapy. This aspect has also been highlighted in previous studies, as it facilitated the transition to VCP (66). Nonetheless, it represents a valuable complement to conventional face-to-face psychotherapy, particularly within a blended therapy model that may combine both modalities (19, 88, 89).

Consistent with findings from previous studies, the UTAUT model demonstrated its high predictive value for explaining the acceptance of eHealth interventions (47, 48, 59, 60, 62, 69). The models' core predictors PE, EE, and SI each exhibited significant effects on the acceptance of VCP, explaining a high level of variance. Among these, PE appeared to be the strongest predictor, as could be reported in previous studies, underscoring its central role for acceptance, matching the respondents' concern of less efficacy of VCP (48, 49, 60, 61). Similarly, the impact of SI highlights the potential to enhance adoption rates through strategic involvement of important relationships to relatives, friends, and caregivers, who could actively promote the use of VCP (62). The notable influence of EE further emphasizes the relevance of user-friendly technology, particularly for individuals with limited prior experience with ICT (46, 69).

Lastly, VCP may not only benefit individuals affected by mental health disorders in Germany, but also contributes to address the global treatment gap in mental health care, particularly in low- and middle-income countries (90). Several international studies have already demonstrated positive attitudes toward VCP, especially with regard to accessibility and flexibility. A literature review focusing primarily on studies from North America (including rural areas) and Australia highlights advantages such as cost savings, discretion, and reduced stigma, while also pointing to barriers like possible technical difficulties and challenges in nonverbal communication—similar to those reported in the present study (53). Consistent with these findings, research from the United Kingdom and Canada reports high levels of satisfaction with VCP (52, 54). A study from Singapore further emphasizes the importance of SI and PE as central predictors of acceptance, while also identifying technology-related issues as potential barriers (55). This international comparison illustrates that e-mental health services have already been implemented across various countries, showing similar drivers and barriers for acceptance. It also highlights that acceptance is influenced not only by individual attitudes but also by broader infrastructural and contextual factors.

With regard to the study objectives, the findings indicate a moderate to high level of acceptance of VCP. Significant drivers of acceptance included present depressive symptoms, current outpatient psychotherapy, digital confidence, as well as the three key predictors of the UTAUT model: PE, EE, SI. These drivers should be specifically supported to enhance the adoption and practical implementation of VCP. Although not statistically significant, factors like Internet anxiety and digital overload also appeared to be relevant for acceptance and need to be considered. Major concerns and thus barriers were related to the expected effectiveness of VCP and the ability to convey emotional expression via video. These factors need to be addressed in order to increase acceptance and promote broader use of VCP. Medical, psychotherapeutic anamnesis and ICT-related data emerged as relevant contributors to acceptance, whereas sociodemographic data appeared to be less influential in this sample. Participants' willingness to conduct nearly 50% of their therapy online demonstrates a readiness for this digital complement in mental health care, what supports a broader implementation. Taken together, these results support the potential of VCP as a valuable supplement to psychotherapeutic care to address the global treatment gap, highlighting aspects that need to be considered to ensure that everyone can benefit from these digital solutions.

## 5 Limitations

Given that the study was conducted through an online questionnaire, individuals lacking internet access or the requisite devices may have been excluded, potentially leading to a biased sample. This exclusion disproportionately affects older individuals and those with lower levels of education, whereas the majority of respondents in this study belonged to higher educational levels (91, 92). Furthermore, individuals with higher levels of digital confidence and lower internet anxiety were more likely to engage with eHealth interventions, potentially increasing their willingness to use VCP. This may cause a limited generalizability of the findings to the broader population. Additionally, as approximately half of the participants had no current or prior experience with psychotherapy, their responses may have been influenced by a lack of familiarity with its contexts. Moreover, the intention-behavior-gap, which refers to the phenomenon that the intention to do something does not necessarily translate into actual behavior, must be considered in this context, as acceptance was operationalized as behavioral intention (93, 94). Since slightly more than half of the participants were previously uninformed about VCP, and half had no prior therapy experience, there is no data about the actual usage behavior to date. Additionally, all responses were self-reported, making them susceptible to common method variance (95, 96). This issue should be mitigated by ensuring the anonymity of the survey and achieving high internal reliability of the included items. Internal consistency for SI was rather low (Cronbach's *α* = .68), indicating that items used may not adequately represent the construct. SI consisted of 2 items. One explanation might be that a lower level of items is associated with Cronbach's *α* values (97). As an adapted version of the UTAUT questionnaire was used, the SI items are in need of improvement in future studies. To address the mentioned limitations, future studies should include a larger number of individuals with lower educational levels as well as more participants who are currently or have previously been in treatment. Additionally, it should be examined whether there are differences in acceptance among individuals with visual or hearing impairments, as this was not specifically investigated in the present study and may pose a potential barrier. Future research should also focus on examining actual usage behaviors and patient adherence to VCP, to overcome the intention-behavior-gap.

## 6 Conclusion

To conclude, the study revealed a moderate to high level of acceptance among patients affected by mental health disorders and healthy controls toward VCP. Significant barriers and concerns, including a perceived reduced effectiveness of VCP and difficulties with emotional expressiveness via video need to be addressed first to meet the needs of all potential users. Targeted trainings, trial lessons and improved information on VCP could be used to increase acceptance. Thereby, drivers, like the high levels of SI and PE and the digital confidence identified here, should be emphasized and supported to promote greater adoption of VCP. Moreover, additional significant drivers as present depressive symptoms and current outpatient psychotherapy should be acknowledged and specifically addressed. Even non-significant factors such as Internet anxiety or digital overload require further consideration, e.g., through the use of user-friendly video platforms and improved data security, as they may become relevant barriers if left unaddressed. Overall, the study suggests that acceptance is influenced by a combination of individual characteristics (e.g., medical, psychotherapeutic anamnesis and ICT-related data) and theoretical predictors derived from the UTAUT model. In light of the present findings, further implementation of VCP, particularly within the context of outpatient psychotherapy, should be considered as a viable complement to face-to-face psychotherapy to enhance future therapeutic processes and improve global psychotherapeutic care provision.

## Data Availability

The data that support the findings of this study are available from the corresponding author upon reasonable request.
