# Supplementary material for: Videoconferencing psychotherapy: determining acceptance, drivers and barriers of use
Source: Front Digit Health. 2025 Aug 29;7:1634013. doi: 10.3389/fdgth.2025.1634013 (PMC12427252; doi:10.3389/fdgth.2025.1634013)
Supplement: Supplementary file 1 [file Datasheet1.docx]

**SUPPLEMENTARY MATERIALS**

**Videoconferencing psychotherapy: Determining acceptance, drivers and barriers of use**

Angelina Nurtsch^1,2^, Lisa Maria Jahre^1,2^, Julia Barbara Krakowczyk^1,2^, Anita Robitzsch^1,2^, Martin Teufel^1,2^, Alexander Bäuerle*^1,2^

^1^Clinic for Psychosomatic Medicine and Psychotherapy, LVR-University Hospital, University of Duisburg-Essen, Essen, Germany.

^2^Center for Translational Neuro- and Behavioral Sciences (C-TNBS), University of Duisburg-Essen, Essen, Germany.

Corresponding Author: PD Dr. Alexander Bäuerle ([alexander.baeuerle@uni-due.de](mailto:alexander.baeuerle@uni-due.de))

**Table SM1**

*Checklist for Reporting Results of Internet E-Surveys (CHERRIES) (1)*

| **Checklist Item** | **Explanation** | **Section** |
| --- | --- | --- |
| **Design** | | |
| Describe survey design | Describe target population, sample frame. Is the sample a convenience sample? (In “open” surveys this is most likely.) | Methods > Design and study population |
| **IRB (Institutional Review Board) approval and informed consent process** | | |
| IRB approval | Mention whether the study has been approved by an IRB. | Methods > Design and study population |
| Informed consent | Describe the informed consent process. Where were the participants told the length of time of the survey, which data were stored and where and for how long, who the investigator was, and the purpose of the study? | Methods > Design and study population |
| Data protection | If any personal information was collected or stored, describe what mechanisms were used to protect unauthorized access. | No personal information was collected or stored during the study. All data were anonymized, ensuring that participants' identities could not be traced. |
| **Development and pre-testing** | | |
| Development and testing | State how the survey was developed, including whether the usability and technical functionality of the electronic questionnaire had been tested before fielding the questionnaire. | Methods > Design and study population |
| **Recruitment process and description of the sample having access to the questionnaire** | | |
| Open survey versus closed survey | An “open survey” is a survey open for each visitor of a site, while a closed survey is only open to a sample which the investigator knows (password-protected survey). | Methods > Design and study population |
| Contact mode | Indicate whether or not the initial contact with the potential participants was made on the Internet. (Investigators may also send out questionnaires by mail and allow for Web-based data entry.) | Methods > Design and study population |
| Advertising the survey | How/where was the survey announced or advertised? Some examples are offline media (newspapers), or online (mailing lists – If yes, which ones?) or banner ads (Where were these banner ads posted and what did they look like?). It is important to know the wording of the announcement as it will heavily influence who chooses to participate. Ideally the survey announcement should be published as an appendix. | Methods > Design and study population |
|  |  |  |
| **Survey administration** | | |
| Web/E-mail | State the type of e-survey (eg, one posted on a Web site, or one sent out through e-mail). If it is an e-mail survey, were the responses entered manually into a database, or was there an automatic method for capturing responses? | Methods > Design and study population |
| Context | Describe the Web site (for mailing list/newsgroup) in which the survey was posted. What is the Web site about, who is visiting it, what are visitors normally looking for? Discuss to what degree the content of the Web site could pre-select the sample or influence the results. For example, a survey about vaccination on a anti-immunization Web site will have different results from a Web survey conducted on a government Web site | Methods > Design and study population |
| Mandatory/voluntary | Was it a mandatory survey to be filled in by every visitor who wanted to enter the Web site, or was it a voluntary survey? | Not applicable |
| Incentives | Were any incentives offered (eg, monetary, prizes, or non-monetary incentives such as an offer to provide the survey results)? | Methods > Design and study population |
| Time/Date | In what timeframe were the data collected? | Methods > Design and study population |
| Randomization of items or questionnaires | To prevent biases items can be randomized or alternated. | Methods > Design and study population |
| Adaptive questioning | Use adaptive questioning (certain items, or only conditionally displayed based on responses to other items) to reduce number and complexity of the questions. | Methods > Design and study population |
| Number of Items | What was the number of questionnaire items per page? The number of items is an important factor for the completion rate. | Methods > Design and study population |
| Number of screens (pages) | Over how many pages was the questionnaire distributed? The number of items is an important factor for the completion rate. | Methods > Design and study population |
| Completeness check | It is technically possible to do consistency or completeness checks before the questionnaire is submitted. Was this done, and if “yes”, how (usually JAVAScript)? An alternative is to check for completeness after the questionnaire has been submitted (and highlight mandatory items). If this has been done, it should be reported. All items should provide a non-response option such as “not applicable” or “rather not say”, and selection of one response option should be enforced. | Not applicable. Non-complete surveys were included, as long as data for the primary outcome (acceptance) was available. |
| Review step | State whether respondents were able to review and change their answers (eg, through a Back button or a Review step which displays a summary of the responses and asks the respondents if they are correct). | Methods > Design and study population |
| **Response rates** | | |
| Unique site visitor | If you provide view rates or participation rates, you need to define how you determined a unique visitor. There are different techniques available, based on IP addresses or cookies or both. | The platform Unipark (Tivian XI GmbH) identifies unique visitors using cookies. |
| View rate (Ratio of unique survey visitors/unique site visitors) | Requires counting unique visitors to the first page of the survey, divided by the number of unique site visitors (not page views!). It is not unusual to have view rates of less than 0.1 % if the survey is voluntary. | This data is not accessible to the study team. |
| Participation rate (Ratio of unique visitors who agreed to participate/unique first survey page visitors) | Count the unique number of people who filled in the first survey page (or agreed to participate, for example by checking a checkbox), divided by visitors who visit the first page of the survey (or the informed consents page, if present). This can also be called “recruitment” rate. | The authors cannot retrieve initial page hits to the survey on Unipark. Consequently, the recruitment rate can not be calculated. The number of participants who provided consent is reported in the Methods section (>Methods > Design and study population). |
| Completion rate (Ratio of users who finished the survey/users who agreed to participate) | The number of people submitting the last questionnaire page, divided by the number of people who agreed to participate (or submitted the first survey page). This is only relevant if there is a separate “informed consent” page or if the survey goes over several pages. This is a measure for attrition. Note that “completion” can involve leaving questionnaire items blank. This is not a measure for how completely questionnaires were filled in. (If you need a measure for this, use the word “completeness rate”.) | Methods > Design and study population |
|  |  |  |
| **Preventing multiple entries from the same individual** | | |
| Cookies used | Indicate whether cookies were used to assign a unique user identifier to each client computer. If so, mention the page on which the cookie was set and read, and how long the cookie was valid. Were duplicate entries avoided by preventing users access to the survey twice; or were duplicate database entries having the same user ID eliminated before analysis? In the latter case, which entries were kept for analysis (eg, the first entry or the most recent)? | Unique users were identified through cookies, with avoiding duplicate entries by restricting users from assessing the survey more than once. |
| IP check | Indicate whether the IP address of the client computer was used to identify potential duplicate entries from the same user. If so, mention the period of time for which no two entries from the same IP address were allowed (eg, 24 hours). Were duplicate entries avoided by preventing users with the same IP address access to the survey twice; or were duplicate database entries having the same IP address within a given period of time eliminated before analysis? If the latter, which entries were kept for analysis (eg, the first entry or the most recent)? | The authors do not have access to the IP adresses of users of the platform Unipark (Tivian XI GmbH). |
| Log file analysis | Indicate whether other techniques to analyze the log file for identification of multiple entries were used. If so, please describe. | No log file analysis was conducted. |
| Registration | In “closed” (non-open) surveys, users need to login first and it is easier to prevent duplicate entries from the same user. Describe how this was done. For example, was the survey never displayed a second time once the user had filled it in, or was the username stored together with the survey results and later eliminated? If the latter, which entries were kept for analysis (eg, the first entry or the most recent)? | Not applicable (open survey). |
| **Analysis** | | |
| Handling of incomplete questionnaires | Were only completed questionnaires analyzed? Were questionnaires which terminated early (where, for example, users did not go through all questionnaire pages) also analyzed? | Methods > Design and study population  Results > Acceptance of videoconferencing psychotherapy and its predictors |
| Questionnaires submitted with an atypical timestamp | Some investigators may measure the time people needed to fill in a questionnaire and exclude questionnaires that were submitted too soon. Specify the timeframe that was used as a cut-off point, and describe how this point was determined. | Methods > Design and study population |
| Statistical correction | Indicate whether any methods such as weighting of items or propensity scores have been used to adjust for the non-representative sample; if so, please describe the methods. | Not applicable |
|  |  |  |

**Study questionnaire**

*Note.* All items were originally presented in German and have only been translated for publication.

*1. Sociodemographic data*

Please indicate your age in years:

Please indicate your gender:

- Female
- Male
- Diverse

Please indicate your current marital status:

- Single
- In a relationship
- Married
- Divorced/separated
- Widowed
- Other (please specify)

Please indicate your highest level of education:

- No educational degree / Other
- Lower secondary education
- Higher secondary education
- Higher education entrance qualification
- University education

Please indicate your current employment status:

- Still in education (e.g. school, university)
- Not employed (e.g. jobseeking, occupational disability)
- Part-time employed
- Employed
- Retired
- Other (please specify)

Are you currently unfit to work (on medical grounds)?

- Yes, since:
- No

Please indicate the type of city or municipality you live in:

- Large city (> 100,000 residents)
- Medium-sized town (> 20,000 residents)
- Small town (> 5,000 residents)
- Rural area (< 5,000 residents)

Do you suffer from a physical or mental health condition that affects your mobility (e.g., reaching your general practitioner, running important errands, etc.)?

- Yes
- No

*2. Psychotherapeutic anamnesis*

Have you been diagnosed with a mental health disorder (by a physician or psychotherapist), currently or in the past?

- Yes, with one mental disorder (please specify)
- Yes, with multiple mental disorders (please specify)
- No

Are you currently using one or more of the following support services?

(Multiple answers possible)

[Responses: currently, in the past, currently and in the past, never.]

- Psychiatric outpatient clinic (institutional setting)
- Psychiatric inpatient psychotherapy
- Psychosomatic inpatient psychotherapy
- Outpatient psychotherapy
- Psychiatric medication
- Socio-psychiatric service
- Other (please specify):

On average, how often do you attend outpatient psychotherapy sessions per month?

[Responses could be given on a scale from 0 to 12 or more]

*3. eHealth data*

[Digital confidence]

How confident are you in dealing with...?

[Responses: very unsure, rather unsure, neutral, rather sure, very sure.]

... digital media?

... platforms on the internet?

... digital devices (e.g. computers)?

… platforms for video-based communication?

How many hours per day do you typically use the Internet for...?

[Responses: not at all, less than 1 hour, 1 to 3 hours, 3 to 5 hours, more than 5 hours.]

… private purposes?

… professional purposes?

How many hours per day do you typically use video-based communication for...?

[Responses: not at all, less than 1 hour, 1 to 3 hours, 3 to 5 hours, more than 5 hours.]

… private purposes?

… professional purposes?

[Digital overload]

To which extent does each of the following statements apply to you?

[Responses: 1 = does not apply, 5 = fully applies]

- I feel stressed by the constant availability via mobile phone or e-mail.
- I feel pressured by unwanted messages and e-mails.
- I feel uncomfortable constantly carrying a mobile device.

[Internet anxiety]

To which extent does each of the following statements apply to you?

[Responses: 1 = does not apply, 5 = fully applies]

- I have concerns about using the Internet.
- I am afraid I might make an irreversible mistake while using the Internet.
- The Internet is something that makes me feel uncomfortable.

[Prior knowledge]

What do you already know about digital approaches of psychotherapy?

[Responses: 1 = strongly disagree, 5 = strongly agree]

- I understand what this means.
- I know how such programs work.
- I know how to ﬁnd such programs.

*4. Acceptance: Unified Theory of Acceptance and Use of Technology questionnaire – adapted for videoconferencing psychotherapy (2)*

[Responses: 1 = does not apply, 5 = fully applies; subscales: BI = behavioral intention, SI = social influence, PE = performance expectancy, EE = effort expectancy]

1. I would like to try videoconferencing psychotherapy. (BI)
2. I would use videoconferencing psychotherapy if it was offered to me. (BI)
3. I would recommend videoconferencing psychotherapy to acquaintances with a mental disorder. (BI)
4. People close to me would approve of the use of videoconferencing psychotherapy. (SI)
5. My general practitioner would approve the use of videoconferencing psychotherapy. (SI)
6. Videoconferencing psychotherapy could improve my general well-being. (PE)
7. The use of videoconferencing psychotherapy would enable me to save time (e.g. for travel). (PE)
8. The use of videoconferencing psychotherapy would enable me to save costs (e.g. for fuel). (PE)
9. Use of videoconferencing psychotherapy could improve my mental health status. (PE)
10. The use of videoconferencing psychotherapy would allow me to better incorporate an appointment into my daily routine. (EE)
11. The use of videoconferencing psychotherapy would enable me to speak to the psychotherapist more openly from a safe environment (e.g. from home). (EE)
12. The use of videoconferencing psychotherapy would not be an additional burden for me. (EE)
13. Videoconferencing psychotherapy would be easy for me to use and understand. (EE)
14. The use of videoconferencing psychotherapy could be incorporated into my daily routine. (EE)

*5. eHealth literacy: German version of the eHealth Literacy Scale (GR-eHEALS) (3)*

*6. eHealth data 2*

Are you aware of the possibility to communicate with a psychotherapist via video consultation?

- Yes
- No

Does your attending psychotherapist already have the option to use videoconferencing psychotherapy?

- Yes
- No
- I don‘t know
- Not in treatment (currently or in the past)

How often have you used digital psychotherapy via video so far?

[Responses could be given on a scale from 0 times to 10 times or more]

In which context would you use videoconferencing psychotherapy?

[Multiple answers possible]

- First consultation
- Pharmacological adjustment
- Medical prescription/certificate of incapacity for work
- Outpatient psychotherapy
- Inpatient psychotherapy
- Partial inpatient psychotherapy
- Psychiatric outpatient clinic (institutional setting)
- Final consultation
- I wouldn't use digital psychotherapy via video for any of these purposes.
- Other (please specify):

To what extent (in %) would you be willing to conduct a videoconferencing psychotherapy?

[Responses could be given on a scale from 0 to 100 %]

How important is it to establish a relationship of trust with your psychotherapist before using a videoconferencing psychotherapy?

- Very important
- Rather important
- Neutral
- Rather unimportant
- Not at all important

For each of the following statements, please rate the extent to which this concern would hinder you from using videoconferencing psychotherapy?
[Responses: 1 = strongly disagree, 5 = strongly agree]

- I don’t have the technical requirements for videoconferencing psychotherapy.
- I am not confident in using the technology required for videoconferencing psychotherapy.
- I do not have a suitable space for a private conversation.
- I am concerned about a lack of data protection.
- I believe that videoconferencing psychotherapy is less effective than face-to-face therapy.
- I worry that I'll be distracted during the session.
- I worry that my psychotherapist will be distracted during the session.
- Videoconferencing psychotherapy makes it more difficult to maintain professional boundaries.
- With videoconferencing psychotherapy,
- I would be unlikely to understand the non-verbal language (e.g. facial expressions and gestures) of the psychotherapist.
- The psychotherapist would be unlikely to understand my non-verbal language (e.g. facial expressions and gestures) well during videoconferencing psychotherapy.
- I worry that videoconferencing psychotherapy could negatively impact the therapeutic relationship.
- Videoconferencing psychotherapy would prevent me from expressing my emotions and feelings.
- Videoconferencing psychotherapy is frightening for me.

*7. Depression: Patient Health Questionnaire-8 (4)*

**References**

1. Eysenbach G. Improving the quality of Web surveys: the Checklist for Reporting Results of Internet E-Surveys (CHERRIES). J Med Internet Res 2004; 6(3):e34.

2. Venkatesh, Morris, Davis. User Acceptance of Information Technology: Toward a Unified View. MIS Quarterly 2003; 27(3):425.

3. Marsall M, Engelmann G, Skoda E-M, Teufel M, Bäuerle A. Measuring Electronic Health Literacy: Development, Validation, and Test of Measurement Invariance of a Revised German Version of the eHealth Literacy Scale. J Med Internet Res 2022; 24(2):e28252.

4. Kroenke K, Strine TW, Spitzer RL, Williams JBW, Berry JT, Mokdad AH. The PHQ-8 as a measure of current depression in the general population. J Affect Disord 2009; 114(1-3):163–73.
